# Complete plastome of the medicinally important plant, *Alstonia scholaris* (*Apocynaceae*)

**DOI:** 10.1080/23802359.2019.1660277

**Published:** 2019-09-06

**Authors:** Lei Jin, Jin Yang, Changkun Liu, Mengling He

**Affiliations:** a College of Traditional Chinese Medicine, Guangdong Pharmaceutical University, Guangzhou, PR China;; b Key Laboratory for Plant Diversity and Biogeography of East Asia, Kunming Institute of Botany, Chinese Academy of Sciences, Kunming, PR China;; c School of Life Science, Yunnan University, Kunming, PR China;; d Key Laboratory of State Administration of Traditional Chinese Medicine for Production and Development of Cantonese Medicinal Materials, Guangzhou, PR China

**Keywords:** *Alstonia scholaris*, complete plastome, phylogenetic analysis

## Abstract

*Alstonia scholaris* is an evergreen tropical tree with significant medicinal values. To better understand its genetic and genomic profiles, we sequenced and assembled the completed plastome of *A. scholaris*. The plastome is 154,699 bp in length, consisting of a large (LSC, 85,364 bp) and a small single-copy region (SSC, 18,027 bp), which are separated by a pair of inverted repeat regions (IRs, 25,654 bp). It possesses 116 unique genes (82 protein-coding genes, 30 tRNAs, and 4 rRNAs). Phylogenetic analysis suggests that *A. scholaris* is sister to the clade including remaining *Apocynaceae* species.

*Alstonia scholaris* (Linn.) R. Brown (*Apocynaceae*) is an evergreen tree distributed in South China, Indochina, New Guinea, and Austrilia (Li et al. 1995). The species is a medicinal plant traditionally used in China, India, Malaysia, and Thailand for the treatment of diarrhoea, dysentery, malaria, fever, and cardiac, as well as respiratory problems (Singh et al. 2017). Leaves of *A. scholaris*, bearing the Chinese name ‘*Dengtaiye*’, have been used as ethnomedicine to treat chronic respiratory diseases in the Yunnan province of China (Cai et al. 2010). However, little is known about the genetic and genomic profiles of the medicinally important plant, hindering the conservation and management of the germplasm resource. Here, we sequenced and assembled complete plastome of *A. scholaris* using high throughput Illumina sequencing technology.

Healthy and fresh leaves of *A. scholaris* were collected from Gengma, Yunnan, China (23°37′55.78″N, 99°19′18.40″E). Voucher specimen (Y. Ji 2017107) was deposited in the Herbarium of Kunming Institute of Botany, Chinese Academy of Sciences (KUN). Silica gel dried leaf tissues were used to extract total DNA by a modified CTAB method (Yang et al. 2014). Subsequently, we sheared the purified DNA by sonication to generate fragments of 500 bp length for constructing a paired-end library. Illumina libraries were prepared according to the manufacturer’s protocol (Illumina, San Diego, California, USA). Ensuing, paired-end sequencing was performed using the Illumina Hiseq 2000 (Illumina, San Diego, California, USA) sequencing platform at BGI (Wuhan, Hubei, China). The complete plastome sequences of *Catharanthus roseus* (GenBank Accession No. KC_563319) was downloaded as the reference for the plastome assembly following the method described by Jin et al (2018). The annotation of the plastome was performed in Geneious version 10.2.3 (Biomatters Ltd, Auckland, New Zealand) (Kearse et al. 2012). The validated plastome sequences of *A. scholaris* deposited in GenBank under Accession number MN176280.

The *A. scholaris* plastome is 154,699 bp in length and presents a typical quadripartite structure consisting of one large single-copy region (LSC, 85,364 bp), one small single-copy region (SSC, 18,027 bp), and a pair of inverted repeat regions (IRs, 25,654 bp). The overall G/C content in the *A. scholaris* plastome is 37.90%, and the corresponding value for LSC, SSC, and IR region were 36.00%, 31.90%, and 43.30%, respectively. The plastome encodes 133 genes, of those, 116 are unique genes (82 protein-coding genes, 30 tRNAs, and 4 rRNAs). Among these unique genes, 9 protein-coding genes (*atp*F, *ndh*A, *ndh*B, *pet*B, *pet*D, *rpl*16, *rpl*2, *rpo*C1, and *rps*12), and 6 tRNAs (*trn*A-UGC, *trn*G-UCC, *trn*I-GAU, *trn*K-UUU, *trn*L-UAA, and *trn*V-UAC) contain one intron, while three protein-coding genes (*ycf*3, *clp*P, and *rps*12) have two introns.

Eleven *Apocynaceae* complete plastomes were included in the phylogenetic analysis. We used RAxML (Stamatakis 2014) with 1000 bootstraps under the GTRCAT substitution model to reconstruct phylogenetic tree. *Gentiana crassicaulis* (*Gentianaceae*) was used to root the tree. The phylogeny robustly supported that *A. scholaris* is sister to the clade including remaining *Apocynaceae* species (Figure 1).

**Figure 1.**
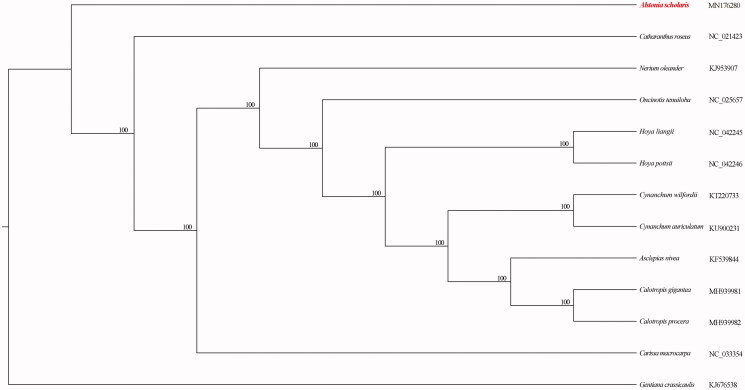
Maximum-likelihood (ML) tree was reconstructed based on thirteen complete chloroplast plastomes. The numbers represent the bootstrap values.
